# Quantitative analysis of intraocular pressure and clinical-morphometric characteristics in Peters’ anomaly: a single-center study

**DOI:** 10.3389/fmed.2025.1520706

**Published:** 2025-06-26

**Authors:** Sebastião Cronemberger, Artur W. Veloso, Maria Valeria C. Pereira, Felipe Leão de Lima, Alexandre Higino Gonçalves da Silva, Luciana de Figueiredo Barbosa, Eduardo Gutemberg Milhomens, Márcio Placedino Martins

**Affiliations:** ^1^Glaucoma Service Professor Nassim Calixto, Hospital São Geraldo, Universidade Federal de Minas Gerais, Belo Horizonte, Brazil; ^2^Anesthesiologist of The Glaucoma Service Professor Nassim Calixto, Belo Horizonte, Brazil

**Keywords:** Peters’ anomaly, intraocular pressure, corneal diameter, axial length, glaucoma

## Abstract

**Purpose:**

To quantitatively analyze intraocular pressure (IOP) alongside clinical and morphometric findings in a cohort of children with Peters’ anomaly (PA).

**Methods:**

This single-center retrospective study included a series of 46 children with PA. Glaucoma diagnosis in a subset was based on abnormal values of IOP, horizontal corneal diameter (HCD), or axial length (AL), individually or combined, compared to pediatric controls utilizing age-specific normative data. Patients were clinically classified into Peters I, II, III, and Peters-plus syndrome. Ultrasound biomicroscopy (UBM) was performed in 10 children.

**Results:**

The cohort of 46 children comprised 27 males and 19 females. All presented with central discoid corneal opacities; 33 cases were bilateral, 13 unilateral. Clinical classifications included 26 Peters I, 10 Peters II, 7 Peters III, and 3 Peters-plus syndrome. In the 29 children analyzed for biometrics, the median age at diagnosis was 7 months (IQR: 1.00–21.00). Median AL was 20.84 mm (IQR: 19.36–22.47 mm) for OD and 20.19 mm (IQR: 18.67–24.05 mm) for OS. Median HCD was 11.5 mm (IQR: 10.50–12.50 mm) for OD and 11.5 mm (IQR: 10.50–13.00 mm) for OS. Glaucoma was diagnosed in 14 patients (48.3%). A significant increase in glaucoma prevalence was observed in children >6 months old (53.6%) compared to those 0–6 months old (26.7%) (*p* = 0.03).

**Conclusion:**

HCD and AL in PA often remain within normal limits, suggesting unique corneal biomechanics. Glaucoma in PA frequently manifests after 6 months of age, necessitating continuous monitoring.

## 1 Introduction

Peters’ anomaly (PA) is a rare, congenital anterior segment dysgenesis characterized by central corneal opacification with a relatively clear periphery. Its estimated incidence is approximately 1.5 per 100,000 births (1, 2). This condition results from abnormal migration of neural crest cells and a disruption of anterior chamber cleavage during the third week of gestation, leading to highly variable anterior chamber anomalies (3). While most cases are sporadic, autosomal recessive and dominant inheritance patterns have been documented, particularly in consanguineous parents (3).

PA is fundamentally characterized by a congenital central opacity within the deep layers of the corneal stroma. The presence or absence of Descemet’s membrane within the posterior corneal ulcer varies. In more severe presentations, annular synechiae of the iris collarette may adhere to the edge of the corneal ulcer. The most advanced form of the anomaly involves the crystalline lens (which may be transparent or opaque) being directly nestled within the corneal ulcer.

Peters-plus syndrome (PpS) represents a distinct entity, characterized by these typical ocular anomalies in conjunction with systemic manifestations. These systemic features include impaired growth, cleft lip and palate, short stature, and developmental delay with varying degrees of intellectual disability (4). PpS typically follows an autosomal recessive inheritance pattern. Despite its etiology remaining largely unknown, the ocular abnormality in PpS has been associated with mutations in genes such as PAX6, PITX2, CYP1B1, and PITX3. A significant concern in most cases of PA is the potential for secondary glaucoma to manifest later in life.

Despite the established link between PA and secondary glaucoma, a comprehensive quantitative investigation evaluating intraocular pressure (IOP) alongside detailed clinical and morphometric findings at the time of initial presentation in pediatric PA patients remains unaddressed in the literature. Such analysis is crucial for understanding the early disease progression and its practical implications for management. Therefore, this study aimed to quantitatively analyze IOP in conjunction with clinical and morphometric findings in a cohort of 46 children diagnosed with Peters’ anomaly.

## 2 Materials and methods

This was a comparative and retrospective study carried out in a single center. The medical records of 46 patients with PA who attended for consultation at the São Geraldo Eye Hospital of the Federal University of Minas Gerais from 1968 to 2024 (56 years) were analyzed. Only patients with documented biomicroscopic findings were included. Demographic data, including race, age, gender, unilateral or bilateral ocular involvement, and parental consanguinity, were evaluated. All the children underwent an ophthalmological examination in the operating room, performed under inhalation anesthesia, which included biomicroscopic assessment, the presence of pannus, posterior embryotoxon, posterior groove, anterior chamber and lens characteristics.

Since 1998, when a DGH5100e echo-biometer (DGH Technology Inc., Exton, PA) became available in our department, AL of 29 (63%) of the 46 children with PA was assessed. Seventeen (37.0%) of the 46 children with PA were excluded from the AL analysis because, until 1997, ultrasound equipment was not available in our department. IOP measurements were taken with a portable applanation tonometer (Draeger tonometer) and HCD measurements with a Jameson calibrator.

All IOP, HCD and AL measurements were performed by the same investigator (SC).

To analyze IOP, HCD and AL, 29 children with PA were stratified according to their age group, compared to age-specific normative data from healthy pediatric controls in two age groups: 0–6 months and >6 months (Table 1) (5).

**Table 1:** Upper limits of normal (97.5th percentile) for intraocular pressure (IOP), horizontal corneal diameter (HCD), and axial length (AL) in healthy children (5).

Age group (months)	# of eyes	IOP (mmHg)	HCD (mm)	AL (mm)
0–6	49	7.1	12.6	21.1
7–12	56	7.3	12.9	22.5
13–24	57	8.8	12.6	22.9
25–36	48	8.1	12.8	23.2
37–48	18	8.8	12.7	25.1
49–60	17	8.8	12.6	23.7
61–79	14	11.8	12.5	23.3

IOP, intraocular pressure; HCD, horizontal corneal diameter; AL, axial length; SD, standard deviation.

The diagnosis of glaucoma in the 29 children with PA at the initial examination was established based on abnormal values of IOP, HCD or AL, either individually or in combination. These parameters were compared to the upper limits of normal values derived from healthy pediatric controls, utilizing age-specific normative data and examined under identical conditions (Table 1) (5). When IOP was considered in isolation for a glaucoma diagnosis, a threshold of >21 mmHg was utilized.

### 2.1 Statistical analysis

Pearson’s chi-squared test was used to compare the absolute frequency of eyes of children diagnosed with glaucoma with those classified as normal in two age groups: 0–6 months and >6 months.

Traditionally, PA has been histologically subdivided into three groups: (1) cornea with central leukoma only; (2) cornea with central leukoma and corneo-lenticular touch; and (3) cornea with central leukoma associated with Rieger mesodermal dysgenesis (6).

**Figure 1:**
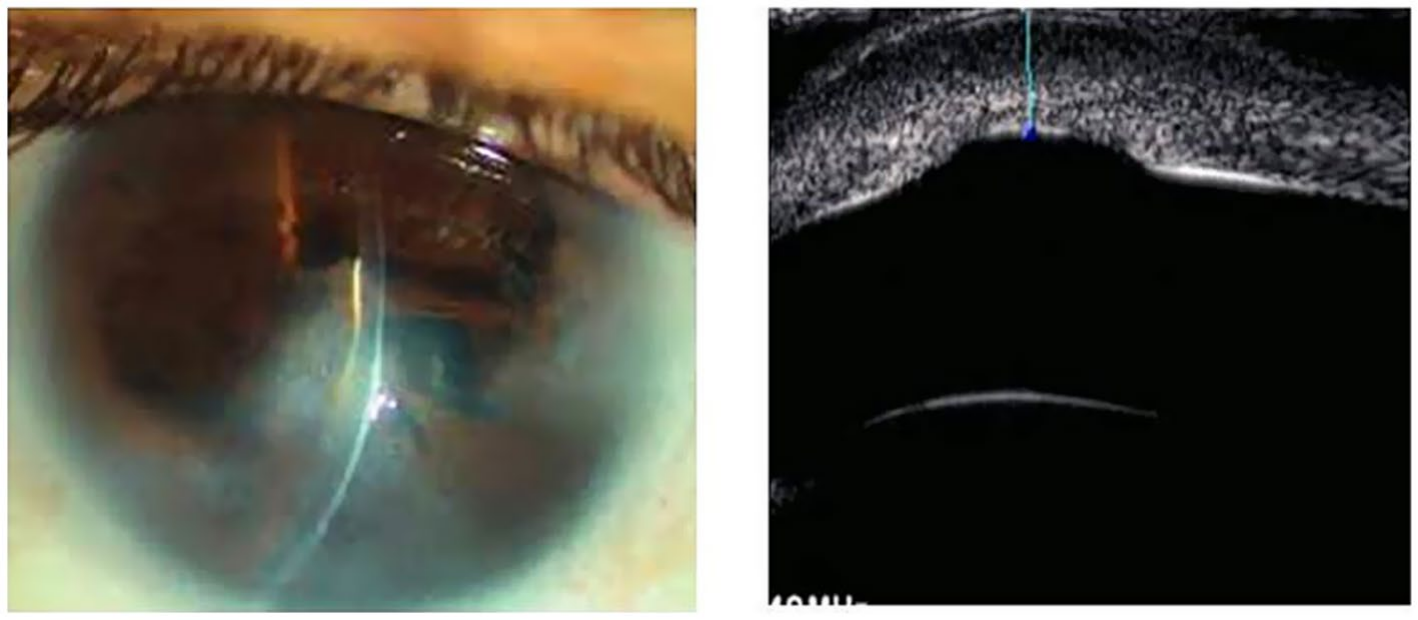
Peters I (corneal opacity) UBM: von Hippel ulcer.

**Figure 2:**
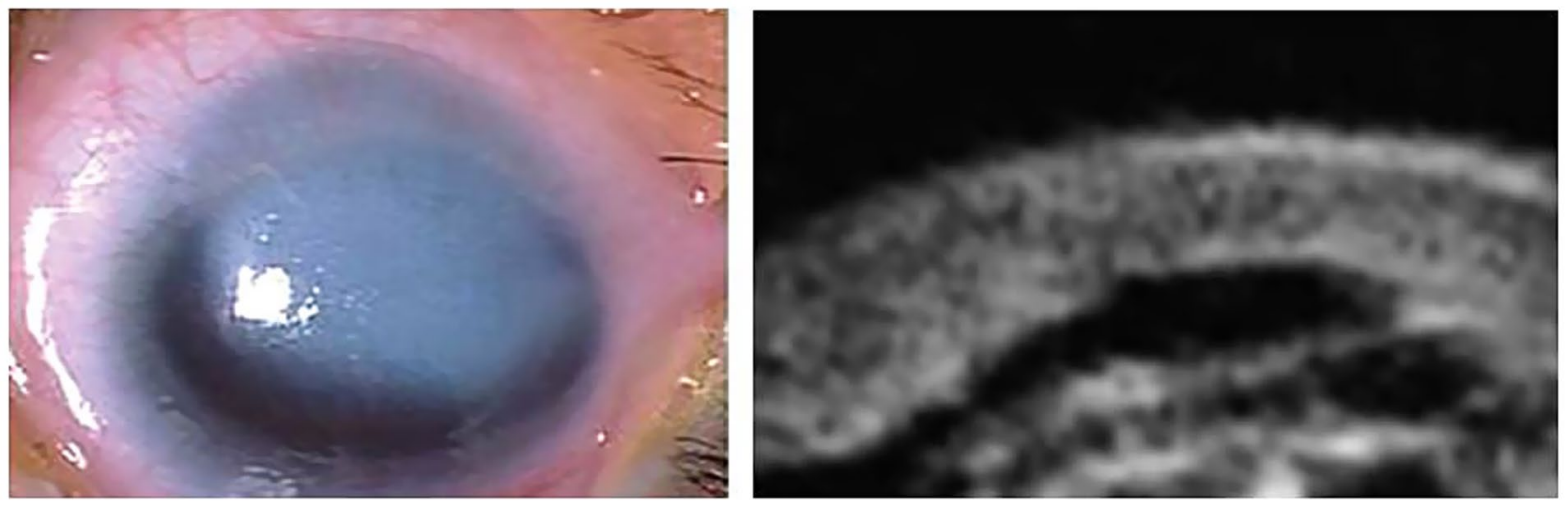
Peters II: corneal opacity with pannus. UBM: corneal opacity plus anterior synechiae at the edge of the ulcer.

**Figure 3:**
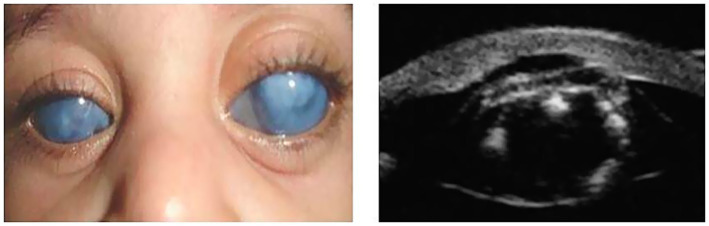
Photo of a child with Peters III: opaque corneas. UBM: showing opaque crystalline attached to the border of the ulcer.

However, for the purpose of this study, a distinct clinical classification system based on biomicroscopic findings was employed, categorizing cases as Peters I, II, III, and PpS. Our classification criteria were as follows: Peters I: Characterized by a posterior corneal ulcer (von Hippel ulcer) (Figure 1); Peters II: Defined by partial or annular synechiae of the iris collarette at the edge of the ulcer (Figure 2); Peters III: Included cases where the crystalline lens (transparent or opaque) was directly nested within the ulcer, in conjunction with Rieger mesodermal dysgenesis (Figure 3).

### 2.2 Imaging modalities

Ultrasound biomicroscopy (UBM) has been reported by Chen et al. (7) as a valuable tool for accurately assessing anterior segment abnormalities in infants with congenital corneal opacities, highlighting its importance in the management of PA with or without Rieger anomalies. Recognizing its utility, our institution has routinely performed UBM in children with primary and secondary congenital glaucoma since 2021. In this study cohort, UBM imaging was successfully obtained in 10 children with PA.

Gonioscopy and ophthalmoscopy were not feasible in this analysis due to significant corneal and/or lenticular opacities.

## 3 Results

The study included 46 children (71 eyes) with PA, 33 (71.7%) with bilateral involvement and 13 (28.3%) with unilateral involvement. Of the cohort, 27 (58.7%) were male and 19 (41.3%) were female. Regarding race, 19 (41.3%) children with PA were identified as White, 17 (36.9%) as Brown, 5 (10.9%) as Black and 5 (10.9%) with undeclared race.

The most significant biomicroscopic findings were corneal opacities with posterior corneal ulcers, annular or partial anterior synechiae, narrow anterior chamber, iris alterations and lens opacities. Based on our clinical classification, the cohort comprised 26 (56.5%) Peters I, 10 (21.7%) Peters II, 7 (15.2%) Peters III and three (6.5%) with PpS. One of the children with PpS had heart disease; the other two were monozygotic twins with an unprecedented association of PpS and aniridia in both eyes associated with bilateral Wilms’ tumor (8).

The demographic and clinical characteristics of the 29 children with PA included for IOP, HCD and AL analysis are presented in detail in Table 2 and summarized in Table 3. The median age at diagnosis was 7 months (interquartile range [IQR], 1.00–21.00 months) with 17 male patients (58.6%) and 12 female patients (41.4%). Race distribution included 12 patients (41.4%) identified as Brown, 10 (34.5%) as White, 2 (6.9%) as Black, and 5 (17.2%) with undeclared race.

**Table 2:** Characterization of 29 patients with Peter’s anomaly.

Age Groups (Months)	Children number	Age (Months)	Gender	Race	AL OD	AL OS	IOP OD	IOP OS	HCD OD	HCD OS	Leukoma	Glaucoma
0–6	1	1	M	Brown	19.41	19.03	4	7	8	10.5	OD	No
	2	3	F	-	18.95	19.51	9	10	12	12.5	BE	No
	3	4	M	Brown	21.61	21.65	32	32	11.75	11.75	BE	Yes (BE)
	4	5	F	Brown	18.73	18.73	20	26	10.5	10.5	BE	Yes (OS)
	5	4	F	White	18.59	18.67	11	6	11.5	11.5	BE	No
	6	1	M	Brown	20.95	20.19	7	7	11	11	BE	No
	7	2	F	Black	21.49	-	15	15	10.5	13	BE	Yes (BE)
	8	2	M	White	20.3	20.11	20	15	12	11.75	BE	No
	9	0	M	White	19.53	19.46	20	6	11	11	BE	No
	10	1	M	White	20.84	20.76	14	14	12	12	BE	No
	11	0	M	-	20.75	-	6	18	13	13.25	BE	Yes (BE)
	12	1	F	Brown	16.55	-	6	5	9	10	OD	No
	13	3	M	Brown	-	-	5	-	11.5	19	BE	Yes (OS)
	14	0	F	-	18.36	18.24	8	8	9.5	9.5	OS	No
	15	1	M	-	19.31	20.3	15	15	11.5	11.5	BE	No
7–12	16	12	M	White	24.75	21.69	8	3	13.5	11.5	BE	Yes (OD)
	17	7	M	Brown	22.47	20.54	13	5	12	11	OD	No
	18	9	M	White	25.59	24.78	15	15	15	15	OD	Yes (BE)
	19	12	F	Brown	22.87	20.97	5	5	11.5	11.5	BE	Yes (OD)
13–24	20	16	M	White	22.53	25.21	20	18	14.5	14.5	BE	Yes (BE)
	21	21	F	Brown	21.83	18.73	2	8	10.75	11.5	OS	No
	22	13	F	White	20.75	19.97	4	4	11.75	11.75	OD	No
25–36	23	25	M	Black	19.04	14.09	2	1	11.25	10.25	OS	No
37–48	24	44	M	-	21.48	18.58	4	4	10.5	9.5	BE	No
49–60	25	52	M	White	26.73	29.03	10	4	14.5	14	OS	Yes (BE)
>60	26	68	M	Brown	31.26	30.88	8	8	15	14	BE	Yes (BE)
	27	122	M	Brown	22.84	24.84	1	5	12.5	12.5	OS	Yes (OS)
	28	69	F	White	24.55	24.4	5	5	14	13	OS	Yes (BE)
	29	69	F	Brown	27.46	29.46	2	0	11	14	BE	Yes (BE)

M, male; F, female; IOP, intraocular pressure; HCD, horizontal corneal diameter; AL, axial length; OD, right eye; OS, left eye; BE, both eyes. Highlighted cells indicate values outside the age-specific normal range or meet the diagnostic criteria for glaucoma.

**Table 3:** Demographic and clinical characteristics of 29 patients with Peters Anomaly.

	Patients, No. (%)
Variable	
Age, median (IQR), months	7 (1.00–21.00)
AL OD, median (IQR), mm	20.84 (19.36–22.47)
AL OS, median (IQR), mm	20.19 (18.67–24.05)
IOP OD, median (IQR), mmHg	8.5 (5.00–16.75)
IOP OS, median (IQR), mmHg	8 (5.00–15.75)
HCD OD, median (IQR), mm	11.5 (10.50–12.50)
HCD OS, median (IQR), mm	11.5 (10.50–13.00)
Gender	
Male	17 (58.6)
Female	12 (41.4)
Race	
Brown	12 (41.4)
White	10 (34.5)
Black	2 (6.9)
Not declared	5 (17.2)
Leukoma	
OD	4 (13.8)
OS	6 (20.7)
Both eyes	19 (65.5)
Glaucoma	
OD	2 (7)
OS	3 (10.3)
Both eyes	9 (31)
Normal	15 (51.7)

IOP, intraocular pressure; HCD, horizontal corneal diameter; AL, axial length; OD, right eye; OS, left eye; BE, both eyes; IQR, interquartile range.

The median AL was 20.84 mm (IQR, 19.36–22.47 mm) for the right eye (OD) and 20.19 mm (IQR, 18.67–24.05 mm) for the left eye (OS). IOP measurements showed a median of 8.5 mmHg (IQR, 5.00–16.75 mmHg) for OD and 8.0 mmHg (IQR, 5.00–15.75 mmHg) for OS. HCD was consistent between eyes, with a median of 11.5 cm (IQR, 10.50–12.50 cm) for OD and 11.5 cm (IQR, 10.50–13.00 cm) for OS.

Leukoma was observed in 19 patients (65.5%) bilaterally, with unilateral leukoma present in 4 patients (13.8%) in the right eye and 6 patients (20.7%) in the left eye. Glaucoma was diagnosed in 14 patients (48.3%), with bilateral involvement in 9 patients (31.0%), right-eye involvement in 2 patients (7.0%), and left-eye involvement in 3 patients (10.3%). Fifteen patients (51.7%) had no evidence of glaucoma at the time of evaluation.

**Figure 4:**
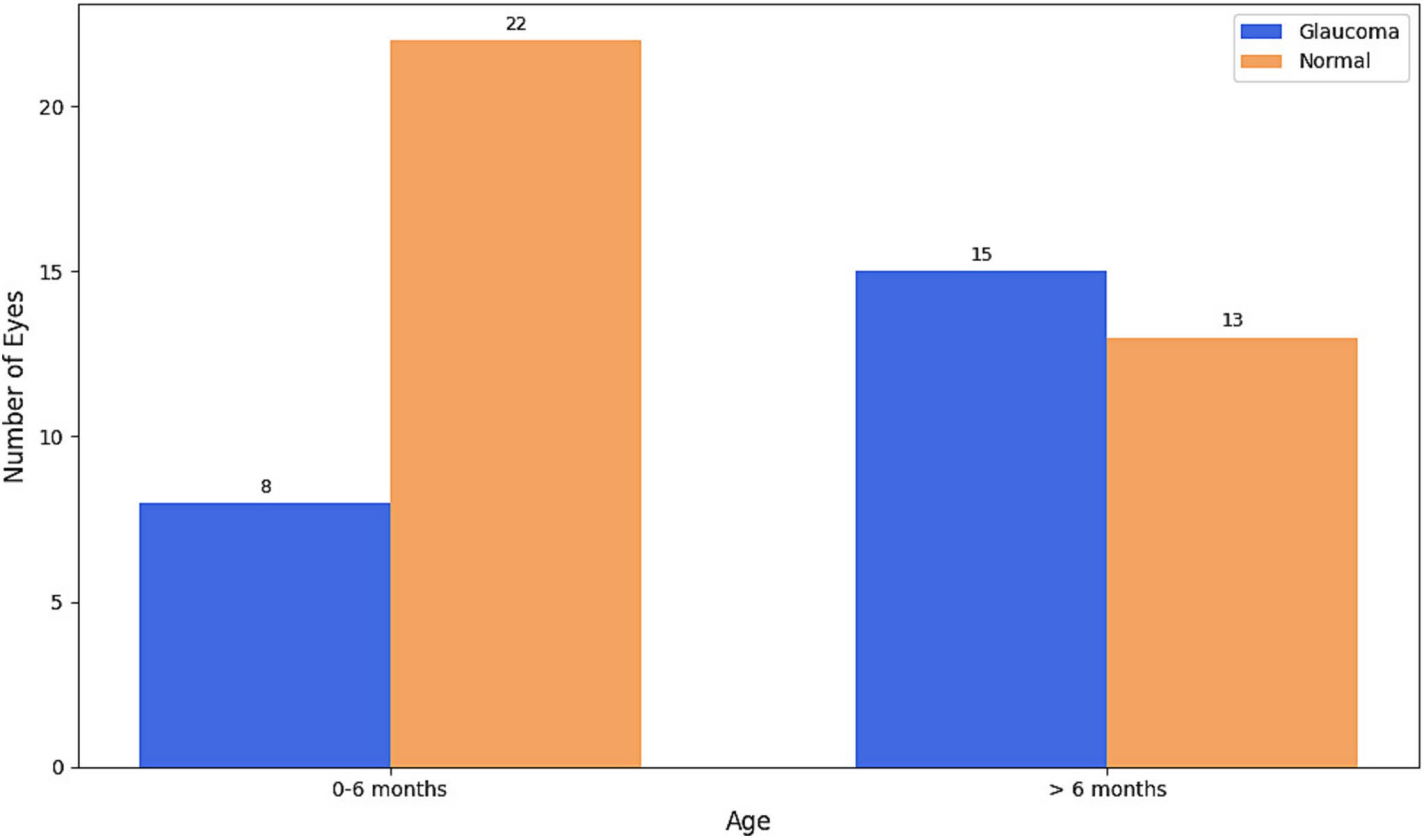
Absolute frequency of eyes of children diagnosed with glaucoma compared to those classified as normal within two age groups: 0–6 months and >6 months.

**Figure 5:**
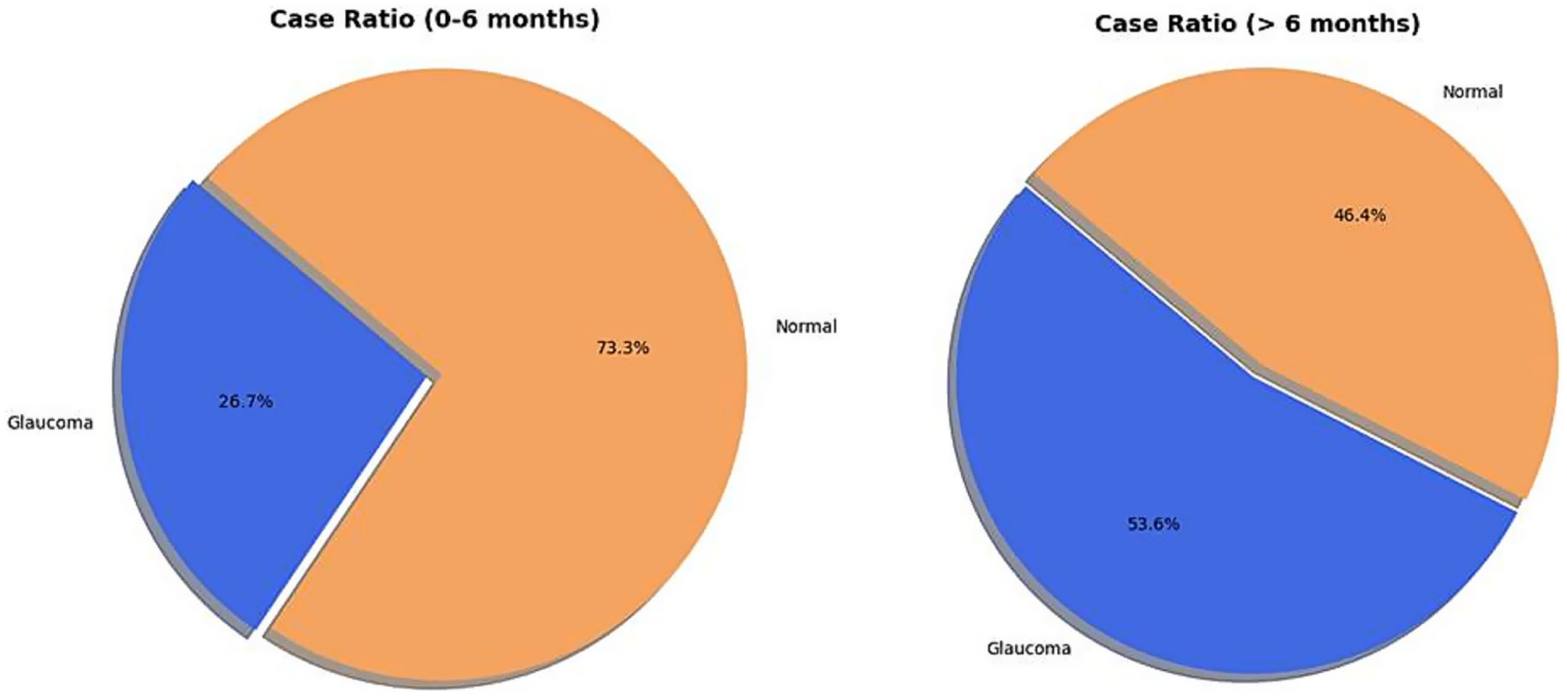
Distribution of eyes of children diagnosed with glaucoma across two age groups: 0–6 months and >6 months.

Of the 29 children analyzed for IOP, HCD, and AL, 15 (51.7%) were aged 0 to 6 months at diagnosis (comprising 30 eyes), while 14 children (48.3%) were over 6 months old (comprising 28 eyes). Glaucoma was present in 8 (26.7%) of the 30 eyes from children aged 0–6 months. In contrast, 15 (53.6%) of the 28 eyes from children older than 6 months had a glaucoma diagnosis (Figure 4). This indicates a statistically significant increase in the relative frequency of glaucoma in patients with PA aged >6 months compared to the 0–6 months age group (Pearson’s chi-squared statistic = 4.38, *p* = 0.03) (Figure 5).

Only three (6.5%) children with PA in this cohort had first-degree consanguineous parents; however, none of these parents exhibited PA. The parents of the two monozygotic twin girls diagnosed with PpS were not consanguineous and did not have PA.

Regarding surgical intervention, five children in this series underwent penetrating keratoplasty (PK) within the last 10 years: one with Peters I, two with Peters II, and two with PpS. The outcomes of these procedures were reported as poor.

## 4 Discussion

PA is a rare congenital corneal opacity, part of a broader group of anterior segment dysgeneses with an estimated prevalence of 3 to 6 per 100,000 individuals (1, 2). Our study, while focused on a single-center cohort, provides valuable insights into the clinical and morphometric characteristics of PA and the incidence of associated glaucoma. Consistent with existing knowledge, the anomaly was predominantly bilateral in our series (71.7%), and we observed a higher frequency in male children.

A key finding of our study pertains to the morphometric characteristics of eyes with PA. Unlike primary congenital glaucoma (PCG), which typically presents a notable increase in HCD and AL during the first 6 months of life (9), a significant proportion of eyes in our PA cohort, even those with glaucoma, exhibited HCD or AL within the normal range.

This phenomenon likely stems from the altered corneal biomechanical properties inherent in PA. We hypothesize that the presence of corneal opacities increases ocular rigidity and reduces corneal hysteresis, thereby impeding the globe enlargement typically observed in response to elevated IOP. This increased corneal resistance may lead to a slower, less pronounced increase in AL, even in the presence of elevated IOP. This observation highlights a fundamental difference in ocular remodeling between PA and PCG.

Our diagnostic criteria for glaucoma in PA also differed from some previous reports. While previous studies (10) primarily relied on an IOP threshold of >21 mmHg, we also defined glaucoma based on abnormal IOP, HCD, or AL values, individually or in combination, compared to pediatric controls utilizing age-specific normative data (Table 1) (5). We believe this comprehensive approach is more robust given the challenges of accurate IOP assessment and fundus examination in children with PA due to corneal opacities. Furthermore, we consider an abnormal AL value to be a particularly reliable indicator of glaucoma, as it is less influenced by factors such as sedation or corneal properties that can confound IOP measurements.

Our data reveal a statistically significant increase in the relative frequency of glaucoma in patients with PA older than 6 months (53.6%) compared to those aged 0–6 months (26.7%, Pearson’s chi-squared statistic = 4.38, *p* = 0.03). This finding underscores that glaucoma in PA is often late-onset, emphasizing the critical need for continuous, long-term observation and proactive management. It is also important to note that secondary glaucoma in PA is often more refractory to medical and surgical treatment than PCG. Given the potential unreliability of IOP assessment in the presence of corneal opacities, we suggest that children with PA who exhibit structural changes, such as an increased HCD or AL, might be considered for prophylactic topical beta-blocker therapy. This recommendation applies even if IOP appears normal at initial presentation, as it aims to mitigate the risk of insidious, late-onset secondary glaucoma in these susceptible eyes.

The primary objective of treating PA is to improve vision and prevent amblyopia. While medical interventions, such as occlusion therapy, can be effective for milder corneal opacities (11–14), surgical intervention is often requisite for visually significant impairment. Penetrating keratoplasty (PK) is considered the definitive surgical approach, typically recommended within the first year of life (15).

Literature indicates that PK outcomes for PA are generally challenging, with reported graft success rates declining significantly over 5 to 10 years post-surgery (16). Factors contributing to graft failure are multifaceted, including anatomical complexities and postoperative complications (16).

Beyond PK, other surgical modalities exist depending on the extent of the anomaly. Peripheral iridectomy can serve as a safer alternative for cases with limited corneal opacity and a clear lens (12–14, 16). Indications for PK typically involve dense, pupil-occluding, or bilateral corneal opacities. Emerging techniques, such as selective endothelial removal, are also being explored for central corneal opacities (17). The prognosis for PA varies significantly based on age at presentation, disease severity (Peters I, II, III), and associated systemic anomalies.” In our cohort, only three (6.5%) children had first-degree consanguineous parents, none of whom exhibited PA. We also describe a unique presentation of PpS in monozygotic twin girls, which, unlike cases reported by Almarzouki et al. (18) and Sawada et al. (19), involved an unusual association with bilateral aniridia and Wilms’ tumor (8). Genetic analysis in these twins revealed six deletions, nine duplications, and 58,558 single nucleotide variants (8). This case further highlights the genetic heterogeneity and phenotypic variability within PA and PpS.

This study has several limitations. It is retrospective in nature and lacks longitudinal follow-up data, which would provide crucial insights into disease progression and long-term treatment outcomes. Another limitation of the present investigation is the absence of direct optic disc imaging data. Due to the prevalent and frequently dense corneal opacities characteristic of Peters’ anomaly, direct fundus visualization and conventional ophthalmic imaging techniques were often precluded. Consequently, glaucoma diagnoses in this study were primarily ascertained based on objective deviations in IOP, HCD, and AL from normative values of pediatric controls categorized by age. While these biometric and tonometric parameters have been reported as indicators of pediatric glaucoma (5, 11), the lack of direct optic disc evaluation implies these diagnoses rely on presumptive criteria derived from surrogate markers. Future investigations leveraging emerging imaging modalities capable of penetrating dense corneal opacities would be invaluable to complement these assessments, offering more comprehensive insights into optic nerve head status. Moreover, a significant strength of this work is that it represents the largest single-center series of PA reported to date, providing a comprehensive characterization of 46 children (71 eyes) from our Glaucoma Service. Furthermore, we have, for the first time, elucidated the distinct morphometric characteristics of eyes with PA, which differ fundamentally from those observed in PCG. The high number of PA cases in our series, compared to the general population incidence, warrants further investigation into potential regional factors or referral biases.

## 5 Conclusion

Our findings indicate that Peters’ anomaly predominantly affects both eyes, with a slight male predominance in our cohort. While corneal opacity is the hallmark of Peters’ anomaly, and increased HCD or AL can serve as diagnostic criteria for glaucoma in some cases, our study demonstrates that these biometric parameters are frequently within normal limits in many eyes, especially during early infancy. This observation is corroborated by the lower incidence of glaucoma diagnoses in our cohort’s children aged <6 months compared to those >6 months, implying unique corneal biomechanical properties that may attenuate or conceal typical globe expansion in early ages. Importantly, our findings demonstrate a significant propensity for late-onset glaucoma in PA, underscoring the necessity for continuous monitoring. Given the challenges of IOP assessment and the generally poor outcomes of PK in PA, particularly in more severe forms, alternative treatment strategies and proactive management are crucial.

## Data Availability

The raw data supporting the conclusions of this article will be made available by the authors, without undue reservation.
